# Intersectional (in) equities in contact coverage of maternal and newborn health services in Nepal: insights from a nationwide cross-sectional household survey

**DOI:** 10.1186/s12889-021-11142-8

**Published:** 2021-06-09

**Authors:** Resham B. Khatri, Yibeltal Alemu, Melinda M. Protani, Rajendra Karkee, Jo Durham

**Affiliations:** 1 School of Public Health, Faculty of Medicine, University of Queensland, Brisbane, Australia; 2 Health Social Science and Development Research Institute, Kathmandu, Nepal; 3 School of Public Health and Community Medicine, BP Koirala Institute of Health Sciences, Dharan, Nepal; 4 School of Public Health and Social Work, Queensland University of Technology, Brisbane, Australia

**Keywords:** Maternal and newborn health, Routine services, Multiple, (dis) advantages, Intersectional analysis, Contact coverage, Continuum of care, Nepal

## Abstract

**Background:**

Persistent inequities in coverage of maternal and newborn health (MNH) services continue to pose a major challenge to the health-care system in Nepal. This paper uses a novel composite indicator of intersectional (dis) advantages to examine how different (in) equity markers intersect to create (in) equities in contact coverage of MNH services across the continuum of care (CoC) in Nepal.

**Methods:**

A secondary analysis was conducted among 1978 women aged 15–49 years who had a live birth in the two years preceding the survey. Data were derived from the Nepal Demographic and Health Survey (NDHS) 2016. The three outcome variables included were 1) at least four antenatal care (4ANC) visits, 2) institutional delivery, and 3) postnatal care (PNC) consult for newborns and mothers within 48 h of childbirth. Independent variables were wealth status, education, ethnicity, languages, residence, and marginalisation status. Intersectional (dis) advantages were created using three socioeconomic variables (wealth status, level of education and ethnicity of women). Binomial logistic regression analysis was employed to identify the patterns of (in) equities in contact coverage of MNH services across the CoC.

**Results:**

The contact coverage of 4ANC visits, institutional delivery, and PNC visit was 72, 64, and 51% respectively. Relative to women with triple disadvantage, the odds of contact coverage of 4ANC visits was more than five-fold higher (Adjusted Odds Ratio (aOR) = 5.51; 95% CI: 2.85, 10.64) among women with triple forms of advantages (literate and advantaged ethnicity and higher wealth status). Women with triple advantages were seven-fold more likely to give birth in a health institution (aOR = 7.32; 95% CI: 3.66, 14.63). They were also four times more likely (aOR = 4.18; 95% CI: 2.40, 7.28) to receive PNC visit compared to their triple disadvantaged counterparts.

**Conclusions:**

The contact coverage of routine MNH visits was low among women with social disadvantages and lowest among women with multiple forms of socioeconomic disadvantages. Tracking health service coverage among women with multiple forms of (dis) advantage can provide crucial information for designing contextual and targeted approaches to actions towards universal coverage of MNH services and improving health equity.

**Supplementary Information:**

The online version contains supplementary material available at 10.1186/s12889-021-11142-8.

## Introduction

In low- and lower-middle-income countries (LMICs), preventable maternal and newborn morbidities and mortalities continue to be major public health problems [1]. There are inequities in maternal and newborn mortalities between countries, and higher mortality rates among disadvantaged groups within countries. An analysis of multi-country data, suggests neonatal mortality among mothers of the lowest wealth quintile declined only marginally compared to mothers of the highest wealth quintile [2]. This suggests, despite overall increases in health services access, survival advantages are disproportionately distributed across different wealth strata.

Most maternal and neonatal deaths can be reduced through the uptake of a range of essential maternal and newborn health (MNH) interventions during pregnancy, childbirth, and the postnatal period. These MNH interventions can be provided during antenatal care (ANC) visits, delivering babies at health facilities assisted by skilled health providers, and postnatal care (PNC) visits within the first month of childbirth [3]. The World Health Organization (WHO) recommends every woman should receive at least 4ANC visits [4], skilled care at birth, and at least three PNC visits during pregnancy-postnatal period [5, 6].

From the life cycle perspective, pregnancy, childbirth, and the postnatal period is considered as MNH continuum of care (CoC). The health of mothers and newborns is interconnected and considered as single entity from their survival perspective. For instance, antenatal interventions contribute to the health of mothers and newborns [7]. The timing of the first ANC visit can influence the uptake of the uptake of subsequent visits (e.g., childbirth in health facilities or PNC visits). Thus, the promoting and monitoring early uptake of ANC is important in supporting women complete the MNH continuum, thereby improved health status of mothers and newborns and reduced disabilities and deaths.

Accessing health facilities for health services is termed contact coverage [8, 9], for instance, health facility visits in pregnancy check-up (e.g., ANC visit). Contact coverage primarily quantifies women’s access to reach health facilities [10] and is considered as a proxy of uptake of recommended health interventions [11]. In most LMICs, there are improving trends of contact coverage of primary health care services, including routine MNH services [12]. However, contact coverage of health services along the CoC is inequitable among different equity dimensions such as gender, ethnicity, education, and socioeconomic position [13]. Conversely, women may have multiple markers of inequity dimensions, for example, woman can be both poor and belong to a disadvantaged ethnic group. These socially disadvantaged identities (e.g., gender, socioeconomic position and ethnicity) can intersect to produce complex patterns of inequity in access to health care and contact coverage across the maternal CoC [14–16] compared to people with more advantaged social identities [17]. Despite this, most research on health services and MNH contact coverage across the CoC, has analysed inequity in contact coverage using single equity indicator, such as socioeconomic position, geographic location or ethnicity [13, 18]. Such unidimensional analyses, however, may hide inequities in contact coverage of health services due to multiple forms of (dis) advantage [19, 20] and are insufficient in understanding coverage of services among women with two or more forms of equity marker.

Increasingly intersectionality, as proposed by theorist Williams Crenshaw in 1990, and which focusses on understanding how the interactions of multiple and interconnected social identities interact to produce inequities [21], is being used in health equity research [15]. Intersectionality proposes different social identities, and processes interact to produce multiple forms of marginalisations [15, 22]. For instance, intersectionality assumes people who are poor and belong to a disadvantaged ethnicity, have experience more disadvantages than those who are poor but belong to an advantaged ethnic group [23]. An underlying assumption of intersectional (dis) advantages is individuals’ social identity and processes associated with power asymmetry [14]. In quantitative population health research, an intersectional perspective relates to the study of strata defined by the combination of several social markers (e.g., wealth status, gender, income), rather than the more conventional analysis using a singular dimension of (dis)advantage. The intersectional analysis therefore supports the ideas of proportionate universalism [24], in which health interventions are combined with the level of disadvantages in specific population groups.

The socioeconomically diverse country like Nepal, there has been good progress in in improving access to health services across the MNH continuum. Over two decades, policies and programs have focused on the improvement of contact coverage of MNH services at the national level in Nepal [25, 26]. For example, the proportion of women with routine MNH visits (e.g., institutional delivery) has increased four-fold from 2001 to 2016 [27–29]. This increase in access is partly explained by government policies incorporating the Safe Delivery Incentive Program (SDIP) [30]. However, persistent inequities in contact coverage of MNH services remain a pressing concern [31]. While institutional delivery assisted by skilled health providers increased from 5% in 2006 to 11% in 2011 among women of the lowest wealth quintile, during the same period, the change among the highest wealth quintile was 58 to 82% [28, 32, 33]. Similar patterns have been observed among women of higher level of education versus illiterate women or women of advantaged ethnicities versus disadvantaged ethnicities [30, 34].

To date most available health equity studies in Nepal have focussed on only one dimension of (dis) advantage, such as wealth status or ethnicity, rather than how different markers of social disadvantage interact to create inequities at different points across the MNH CoC. This research begins to address this gap by applying a novel approach to understanding intersectional inequities in contact coverage across the MNH CoC, a more nuanced understanding of the interactions, and effects of joint inequities on contact coverage. This study also provides the latest nationwide contact coverage status of all three routine MNH indicators among women with multiple forms of (dis) advantages (intersectional strata) across the MNH continuum. The study also develops a composite indicator of intersectional (dis) advantages that can be incorporated into routine health management information system (HMIS) and national surveys such as the Nepal Demographic and Health Survey (NDHS) to monitor equity in MNH services contact coverage, allowing policy-makers to design more targeted interventions.

## Methods

### Data source and sampling design

We conducted a further analysis of secondary data from the Nepal Demographic and Health Survey (NDHS) 2016 [27]. The NDHS 2016 is the fifth round periodic nationally representative survey conducted in every five since 1996.

Participants detail and sampling methodology are described in the NDHS 2016 report [27]. In brief, the NDHS 2016 adopted a two-stage cluster sampling design, with probability proportional to size (PPS) (Supplementary file, Fig. S1). The PPS sampling design is commonly used by LMICs conducting nationally representative surveys. The cluster PPS sampling design captures representative samples from a geographically and ethnolinguistically diverse country context, such as Nepal [35]. In the first stage of NDHS 2016, each province was stratified into urban and rural areas, yielding 14 sampling strata. The rural and urban areas were further divided into wards which are called as primary sampling units (PSUs). If wards (in urban areas) were larger size, ward > 200 households were segmented into sub-wards. Therefore, sub-ward from urban areas and wards from rural areas were considered as ward enumeration areas (EAs). A total of 383 wards, one each from PSU, were selected with a probability proportional to the ward size, with independent selection in each sampling stratum within the allocated strata. Sample wards were primary sampling units (PSUs or clusters) selected independently [27]. In the second stage of the NDHS 2016, a total of 30 households per EA were selected with an equal probability of systematic selection from the household listing [27]. In addition, there were no replacements of, or changes to, the pre-selected households allowed in the implementing stage. The NDHS 2016 sampling weights have been calculated and applied, so results are representative at the national as well as strata levels. This study included 1978 women aged 15–49 years who had a live birth in the two years preceding the survey. The NDHS 2016 collected information on pregnancy, childbirth and postnatal care from women who had a live birth in the two years preceding the survey.

### Conceptual framework of the study

**Fig. 1:**
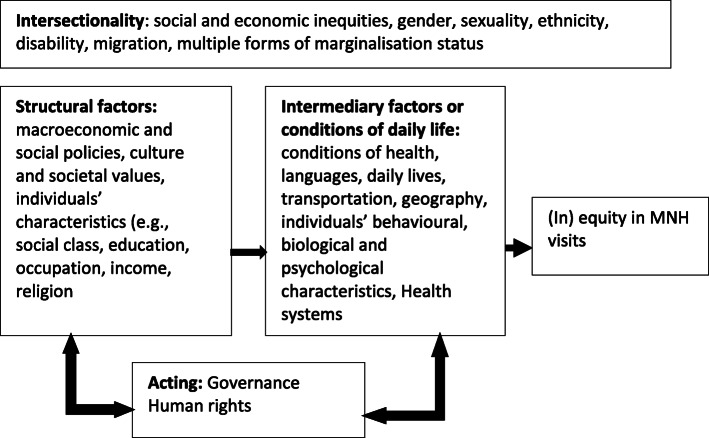
Conceptual framework adapted from Marmot 2018 [36]

Figure 1 shows the conceptual framework used in this study, adapted from Marmot 2018, and modified for this study [36]. The conceptual framework depicts structural factors (e.g., wealth status), and intermediary factors (e.g., geography, transportation) and that are linked with power or oppression [36, 37]. Women may experience intersectional (dis) advantages across these structural and intermediary factors which influence equity of MNH services contact coverage across the healthcare continuum.

### Study variables

Independent variables included socioeconomic and geographic characteristics of women such as ethnicity, wealth status, education, marginalisation status, language, residence, province, and region (Supplementary file, Table S1). Taking reference from past studies [34, 38–40], socioeconomic variables such as ethnicity, level of education of women, wealth status, and marginalisation status of women were further defined. The Government of Nepal has categorised 123 ethnicities into six broader categories [41]: i) Dalits (untouchable), ii) disadvantaged indigenous, iii) disadvantaged non-Dalit Terai caste groups, iv) religious minorities (Muslims), v) relatively advantaged indigenous groups, and vi) upper caste groups (advantaged groups inlcude Brahmin and Chhettri). Taking previous studies [38, 42], these broader categories of ethnicities were merged into two groups according to their comparative privileges. Disadvantaged ethnicities included first four groups (i, ii, iii, and iv) while advantaged ethnicities included later two groups (v and vi). Similarly, women’s education was categorised into illiterate (who cannot read and write), and literate (who can read and write, also and who have primary education or higher). In the NDHS 2016, wealth quintiles were constructed using principal component analysis (PCA) based on more than 40-asset items being owned by households. These wealth quintiles were merged into two groups such as lowest two quintiles as Poor (lower 40%), and upper three quintiles as Rich (upper 60%). A new variable (marginalisation status) was created based on education, wealth status, and ethnicity [43]. Multiple marginalisation status of women (detail in Supplementary file, Table S1) was created based on other three socioeconomic variables included ethnicity (advantaged ethnicity = 1, disadvantaged ethnicity = 0), education (literate = 1, illiterate = 0), and wealth status (rich = 1, and poor = 0). Marginalisation status had eight categories based on the levels of (dis) advantages, for instance, triple forms of disadvantage: women with poor and illiterate and disadvantaged ethnicity; three categories with each of double of forms of disadvantage (women who were poor and illiterate and advantaged ethnicity; women who were poor and literate and disadvantaged ethnicity; women who were rich and illiterate and disadvantaged ethnicity); three categories with each of one form of disadvantage (women who were poor and literate and advantaged ethnicity; women who were rich and illiterate and advantaged ethnicity; women who were rich and literate and disadvantaged ethnicity), and triple advantages: women who were rich and literate and advantaged ethnicity.

This study had three outcome variables specific to contact coverage of MNH visits: i) uptake of 4ANC, ii) institutional delivery and iii) at least one PNC within for mothers and newborn within 48 h of childbirth. Each outcome variable was dichotomised into ‘yes’or ‘no’, based on women’s response in the recorded in the survey, for example, uptake of 4ANC: ‘yes’ or ‘no’. Unlike previous studies on separate PNC visit for mothers [27, 38] and newborns [27], this study created a combined contact coverage of PNC visit for mother-newborn pair.

### Statistical analysis

Independent binomial logistic regression analysis was conducted for each outcome variable. Data analysis was adjusted using sampling weights available in the dataset. All analyses were weighted to adjust for the two-stage cluster sampling used in the NDHS 2016 survey (primary sampling unit = 383; stratification (place of residence and province; strata = 14); survey weights (probability weight = sample weight/1,000,000) [27]. *P*-values were set *p* < 0.05 (two-tailed) as the statistical significance level for the independent variables associated with the outcome variable. All weighted estimates were reported (unless otherwise indicated) including frequency, and proportion (%), reported the extent of inequities in coverage of MNH visits/services in terms of odds ratios (OR) with 95% confidence intervals (CIs). As the NDHS 2016 used cluster sampling design, the clustering effect was adjusted using ‘svy’ command of the Stata version 14.0. Before running the multivariable regression analyses, multi-collinearity was checked and excluded independent variables having variation inflation factors ≥3 in the multivariable regression analyses [44]. The multi-collinearity effect of variables was observed, for instance, region was correlated with the province, and ethnicity, education of women, and wealth status were correlated with marginalisation status of women, therefore, were excluded region, ethnicity, education, and wealth status in the final multivariable analysis of each outcome variable. Maternal age [45], and birth order [46] were confounders and adjusted in the final regression analyses for each outcome variable.

Similarly, three socioeconomic variables—ethnicity, women’s education, and wealth status—were also excluded in the analyses due to their multi-collinearity effects with their composite variable “marginalisation status of women”. Backwards elimination multivariable logistic regression analyses were adopted [47]. For this, firstly, a full multivariable regression model was run and then estimated *p*-values for each independent variable, identified the most significant independent variable and estimated the model. Secondly, this procedure was repeated until no significant independent variable was left at *p* < 0.05 [48]. Adjusted odds ratios (aOR) with 95% CIs of all multivariable model with three outcome variables were reported. The goodness of fit tests was conducted using the Hosmer Lemeshow test (non-significant results (*p* > 0.05) indicated an adequate fit) for binomial logistic regressions [49]. All analyses were conducted using Stata 14.0 (Stata Corp, Texas, USA, 2015).

## Results

Table 1 shows the background characteristics of women included in this study. Among the 1978 women, 42% were from households in the lowest two wealth quintiles as measured by the wealth quintile used in the NDHS. More than two-thirds (69%) of women were from disadvantaged ethnic groups, mostly Madhesi, Janajatis and Dalits. One in ten women (10%) were classified to have all three markers of disadvantage (illiteracy, disadvantaged ethnicities, and lower wealth status). Nearly two in five women (42%) were native Nepali speakers (the national language).

**Table 1:** Socioeconomic and geographic characteristics of respondents women in NDHS 2016

Determinants	Frequency (*N* = 1978)	%
Structural		
Wealth rank		
Poor (40%)	832	42.3
Rich (60%)	1146	58.2
Ethnicity		
Disadvantaged	1374	69.8
Advantaged	604	30.7
Education		
Illiterate	570	29.0
Literate	1408	71.5
Intersectionality		
Marginalisation status of women		
Poor and illiterate and disadvantaged ethnicity	197	10.0
or and illiterate and advantaged ethnicity	70	4.0
Poor and literate and disadvantaged ethnicity	347	18.0
Rich and illiterate and disadvantaged ethnicity	283	14.0
Poor and literate and advantaged ethnicity	218	11.0
Rich and illiterate and advantaged ethnicity	20	1.0
Rich and literate and disadvantaged ethnicity	547	28.0
Rich and literate and advantaged ethnicity	296	15.0
Intermediary		
Language		
Nepali	839	42.6
Maithili	360	18.3
Bhojpuri	267	13.6
Others (e.g., Newari or Tharu)	512	26.0
Provinces		
One	338	17.2
Two	513	26.1
Three	312	15.9
Four	164	8.3
Five	364	18.5
Six	121	6.1
Seven	166	8.4
Residence		
Urban	1062	54.0
Rural	916	46.5
Region		
Mountain	131	6.7
Hill	760	38.6
Terai	1087	55.2

More than half (55%) of women were from the Terai (Plain) Region. One in four women (26%) were from province two, whereas the smallest percentage of women (6%) were from province seven. About half (46%) of women were from urban areas.

Table 2 shows patterns of contact coverage of MNH services across different strata of socioeconomic and geographic variables. The national average of contact coverage three outcome variables, i.e., 4ANC visits, institutional delivery, and PNC visit was 71, 64 and 51%, respectively.

**Table 2:** Contact coverage of routine MNH visits in Nepal, NDHS 2016

Determinants	Frequency	4ANC visits (N = 1978)		Institutional delivery (*N* = 1978)		PNC visit (N = 1978)	
		Yes (%)	p	Yes (%)	p	Yes (%)	p
Structural							
Wealth rank							
Poor (40%)	832	64.9	< 0.001	48.7	< 0.001	39.6	< 0.001
Rich (60%)	1146	75.2		75.4		58.4	
Ethnicity							
Disadvantaged	1374	65.7	< 0.001	59.6	< 0.001	44.8	< 0.001
Advantaged	604	82.4		74.7		63.6	
Education							
Illiterate	570	53.5	< 0.001	47.3	< 0.001	36.3	< 0.001
Literate	1408	77.9		71.0		56.3	
Intermediary							
Language							
Nepali	839	80.2	< 0.001	70.2		57.7	< 0.001
Maithili	360	67.6		56.5		40.7	
Bhojpuri	267	45.6		53.0		33.9	
Others (e.g., Newari or Tharu)	512	70.9		65.7		54.3	
Provinces							
One	338	78.8	< 0.001	65.9	< 0.001	54.5	< 0.001
Two	513	58.0		54.7		38.3	
Three	312	75.8		72.4		62.8	
Four	164	75.3		73.5		63.8	
Five	364	74.7		65.6		51.4	
Six	121	54.6		43.7		36.4	
Seven	166	84.0		77.4		52.1	
Residence							
Urban	1062	75.5	0.003	73.6	< 0.001	57.5	< 0.001
Rural	916	65.4		53.3		42.4	
Region							
Mountain	131	71.7	0.015	45.0	0.007	45.6	< 0.001
Hill	760	76.2		67.7		57.1	
Terai	1087	67.0		64.1		46.5	
National level	1978	71.0		64.0		51.5	

Note: p-values obtained from Fisher exact test. ANC: antenatal care. PNC: postnatal care

The contact coverage of 4ANC visits among illiterate women was lower (53%), province six (55%), women who speak Bhojpuri (46%) compared to literate women (78%), and the province seven (84%) and Nepali native speaking women (80%). Only 47% of illiterate women deliver babies at health facilities, compared to 71% of literate women, and women in provinces two and six were 54 and 44% respectively, compared to 74 and 77% in provinces four and seven respectively. Contact coverage of PNC visit was lower among women of disadvantaged ethnicities (45%), women with lower wealth status (40%), or who were illiterate (36%), and in province six (36%) relative to their advantaged counterparts. Only 34% of Bhojpuri speaking women had contact coverage of PNC visit compared to women with Nepali native speaker (58%) (Table 2).

### Descriptive analysis of intersectional (in) equities of MNH services

**Fig. 2:**
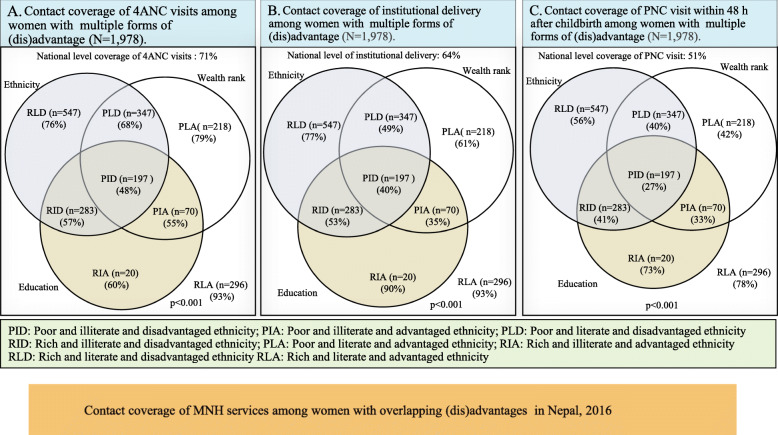
Contact coverage of routine MNH visits among women intersectional (dis) advantages in Nepal, 2016

Importantly, the contact coverage of 4ANC visits, institutional delivery, and PNC visit among women with triple forms of disadantages was 48, 40, and 27% respectively compared to 93, 93 and 78% respectively among women with triple forms of advantages (Fig. 2).

In the bivariable regression analysis, all independent variables (language, marginalisation status, province, place of residence) were significantly associated with each outcome variable. Table 3 shows the results for the multivariable binomial logistic regression analysis of contact coverage of the MNH services. Relative to women with triple disadvantage, the odds of contact coverage of 4ANC visits was more than five-fold higher (aOR = 5.51; 95% CI: 2.85, 10.64) among women with triple advantage. Similarly, women with triple advantage had higher odds (aOR = 7.32; 95% CI: 3.66, 14.63) of institutional delivery compared to women with triple forms of disadvantages. In addition, the odds of uptake of contact coverage of PNC visit among women with triple forms of advantage was four-fold higher (aOR = 4.18; 95% CI: 2.40, 7.28) compared with its reference groups (Table 3). Wealth and ethnic ‘disadvantage’ respectively appear to have a larger association with poor contact coverage (across all three indicators) than literacy. For instance, the odds of contact coverage of institutional delivery and PNC visits among rich, illiterate, advantaged was higher compared to rich, literate, disadvantaged women.

**Table 3:** Multivariable regression analysis of inequity in contact coverage of routine MNH visits in Nepal, 2016 (*N* = 1978)

Determinants	four ANC visits		Institutional delivery		PNC visit	
	Crude OR (95% CI)	Adjusted OR (95% CI)	Crude OR (95% CI)	Adjusted OR (95% CI)	Crude OR (95% CI)	Adjusted OR (95% CI)
Intersectionality						
Marginalisation status of women						
Poor and illiterate and disadvantaged ethnicity	1.00	1.00	1.00	1.00	1.00	1.00
Poor and illiterate and advantaged ethnicity	1.31 (0.71, 2.44)	0.86 (0.45,1.62)	0.83 (0.43, 1.61)	0.65 (0.32, 1.31)	1.35 (0.71, 2.56)	1.41 (0.71, 2.80)
Poor and literate and disadvantaged ethnicity	2.28 (1.44, 3.59) ***	1.38 (0.84, 2.25)	1.46 (0.96, 2.24)	0.70 (0.43,1.15)	1.77 (1.12, 2.79) *	0.96 (0.58, 1.59)
Rich and illiterate and disadvantaged ethnicity	1.43 (0.91, 2.24)	1.54 (0.95, 2.49)	1.72 (1.04, 2.86) *	1.98 (1.10, 3.55) *	1.84 (1.19, 2.84) **	1.96 (1.20, 3.20) **
Poor and literate and advantaged ethnicity	4.15 (2.50, 6.90) ***	1.86 (1.05, 3.26) *	2.43 (1.46, 4.05) ***	1.12 (0.62, 2.03)	2.94 (1.81, 4.79) ***	1.75 (1.03, 2.96) *
Rich and literate and disadvantaged ethnicity	3.39 (2.17, 5.30) ***	2.37 (1.42,3.97) **	5.16 (3.37, 7.91) ***	3.20 (1.97, 5.20) ***	3.45 (2.23, 5.35) ***	2.17 (1.35, 3.51) **
Rich and illiterate and advantaged ethnicity	1.63 (0.30, 8.83)	1.01 (0.21,4.93)	13.28 (2.28, 77.28) **	6.19 (1.08, 35.43) *	7.43 (1.85, 29.90) **	4.35 (1.35, 14.01) *
Rich and literate and advantaged ethnicity	14.34 (7.51, 27.40) ***	5.51 (2.85,10.64) ***	20.19 (10.45, 39.03) ***	7.32 (3.66,14.63) ***	9.63 (5.88, 15.75) ***	4.18 (2.40, 7.28) ***
Intermediary						
Language						
Nepali	1.00	1.00	1.00		1.00	
Maithili	0.51 (0.35, 0.75) ***	0.75 (0.40, 1.41)	0.55 (0.37, 0.82) **		0.50 (0.35, 0.71) ***	
Bhojpuri	0.21 (0.14, 0.31) ***	0.26 (0.13, 0.52) ***	0.48 (0.31,0.74) ***		0.38 (0.25, 0.55) ***	
Others (e.g., Newari or Tharu)	0.60 (0.43,0.85) **	0.62 (0.41, 0.92) *	0.81 (0.58, 1.15)		0.87 (0.66, 1.16)	
Provinces						
One	1.00	1.00	1.00	1.00	1.00	1.00
Two	0.37 (0.24, 0.57) ***	0.69 (0.41,1.16)	0.62 (0.40,0.97) *	0.77 (0.44, 1.33)	0.52 (0.35, 0.77) **	0.87 (0.54,1.40)
Three	0.84 (0.46, 1.55)	0.78 (0.45, 1.35)	1.36 (0.74, 2.50)	1.11 (0.62, 1.98)	1.41 (0.87, 2.26)	1.19 (0.77,1.85)
Four	0.82 (0.50, 1.34)	0.65 (0.38, 1.09)	1.43 (0.74, 2.78)	1.34 (0.69, 2.60)	1.47 (0.84, 2.55)	1.38 (0.82, 2.32)
Five	0.80 (0.46, 1.37)	1.05 (0.62, 1.78)	0.99 (0.61, 1.60)	0.91 (0.56,1.48)	0.88 (0.58, 1.35)	0.87 (0.58, 1.29)
Six	0.32 (0.21, 0.51) ***	0.34 (0.21, 0.56) ***	0.40 (0.24, 0.68) ***	0.73 (0.40, 1.33)	0.48 (0.31, 0.74) ***	0.62 (0.39, 0.99) *
Seven	1.41 (0.90, 2.20)	1.83 (1.05, 3.21) *	1.77 (0.96, 3.26)	2.79 (1.38, 5.65) **	0.91 (0.57, 1.43)	0.86 (0.55, 1.37)
Residence						
Urban	1.00	1.00	1.00	1.00	1.00	1.00
Rural	0.61 (0.44, 0.85) **	0.91 (0.67–1.23)	0.41 (0.30, 0.56) ***	0.53 (0.39, 0.72) ***	0.54 (0.42, 0.70) ***	0.74 (0.58, 0.95) *

## Discussion

This study examined contact coverage of routine visits across the MNH CoC using an intersectional understanding of inequities. First, we found two-thirds of women in Nepal received 4ANC visits and gave birth at health institutions, and one in two (51%) mother-newborn pair, received one PNC visit. All forms of contact coverage of MNH visits were low among socioeconomically and geographically disadvantaged women. Secondly, women with multiple forms of advantage had higher odds of contact coverage of MNH visits across the continuum, compared to their disadvantaged counterparts. For instance, women of lower wealth status and disadvantaged ethnicity had lower coverage compared to women with a single form of disadvantage. Thirdly, contact coverage of all MNH visits was not uniform across the MNH continuum. The highest contact coverage was for 4ANC visits, with increasing dropout along the MNH continuum. Overall, the results show a trend towards increased inequity of MNH visits across the CoC, confirming previous work in Nepal and other South Asian countries [25, 50].

The study indicates that despite policies and programs in Nepal aimed at improving MNH, including the safe delivery incentive program (SDIP) and the free health care program, affirmative action to disadvantaged women is needed to reduce persistent inequities in contact coverage [51]. Continuing to implement programs using a one-size-fits-all approach, is likely to increase access to services, rather than decrease equity gaps among women with social disadvantages [52–54]. Available evidence suggests when policies and programs are designed and implemented using a universal approach, people within the higher socioeconomic strata of society receive services first compared to disadvantaged women. Once saturation is achieved among privileged groups, then lower segments of the population start to receive health services [54]. Decreasing equity gaps therefore is likely to require targeted and contextual strategies for women with multiple disadvantages.

The study demonstrates how wealth status, level of education and ethnicity intersect and impact on contact coverage of all forms of MNH services confirming the intersectionality theory [55]. As suggested by intersectional theory, women with more disadvantages had lower odds of uptake of MNH services. Disadvantages due to wealth status, access to education and ethnicity are rooted in power over the distribution of resources including income, goods and services [56] and due deep-rooted socio-cultural values and, in Nepal, the continuing caste system [57]. The current study applied the concept of intersectionality in quantitative analysis using nationally representative dataset and developed some methodological and thematic insights. The quantitative intersectional analysis could give the status of access to the health services among people with intersectional (dis) advantages [17]. Identification of coverage of health services among most disadvantaged groups can help to design targeted approaches including monitoring of health services using intersectional indicator.

There might be several reasons for poor coverage of contact for these MNH visits. First, the NDHS 2016 reported about 96% of women had at least one ANC visit, with subsequent visits along the MNH continuum had lower than the first ANC visit. While most women had at least one contact with ANC services, few women completed the MNH continuum, with those women with more disadvantages were more likely to drop out across the MNH continuum. Typically, institutional delivery is more influenced by the location of the health facility and continuous availability of health services. In Nepal, not all rural health posts are accredited as birthing centers, meaning women must travel long distances, often along difficult terrain, to birthing centers or hospitals for childbirth. In many instances, women also face challenges in accessing transport to take them to these facilities. Lower PNC visits than the institutional delivery rate, indicates women are not getting PNC services in the crucial first 24 h after birth even when they deliver in a health facility. Thus, mothers and newborns should get special attention after childbirth or after discharge from health facility to complete at one PNC visit.

Secondly, there is no any incentive for PNC visit in Nepal and for many women in the absence of obvious complications, they do not feel there is a need for PNC [58]. Uptake of PNC is also influenced by sociocultural and religious factors, which may restrict women and newborns from leaving their home until the 11th day of as a means of protecting them from illness [58, 59]. Previous studies have demonstrated nearly equal PNC visits with institutional delivery at the national level [27, 32]. This analysis however, considered combined PNC visit for newborns and mothers and found have lower coverage than previous studies, as the mother and newborn as a single entity, form the health service delivery.

The study also demonstrated the benefits of applying an intersectional lens to quantitative survey data, showing how it can be incorporated into routine health monitoring information systems (HMIS), surveys (e.g., NDHS) and programs. The current HMIS in Nepal however [60] does not monitor contact coverage among women with intersectional (dis) advantages [32, 38]. Yet, as this study reveals, an intersectional analysis can provide decision-makers with critical information for developing and implementing tailored interventions for defined population groups. Finally, intersectional lens can assist decision makers in resource allocation to ensure that no one is left behind. Such an analysis is likely to be crucial in achieving the SDGs and universal healthcare access.

This study has some limitations. First, inferences drawn from this study are based on an observational and cross-sectional design, which allows the study of correlations rather than causality. Future prospective observational studies in this research area should incorporate Directed Acyclic Graphs (DAGs) in their study design/analyses to explore the interaction between these variables and the potential mediating effect of education/wealth between ethnicity and health service access/utilization. Second, the outcome variable was self-reported after face-to-face interviews with women which may have social desirability bias (e.g., over reporting of good behaviours and underreporting of bad behaviours) and misclassification. Fourth, some of the subgroups of a variable (e.g., marginalisation status of women) has small sample size (e.g., rich and illiterate and advantaged ethnicity category has 20 women), there is likely to be a large degree of random error. Subgroups with small sample sizes should be interpreted with caution due to the lack of precision and the magnitude of these effect estimates need to be confirmed in future studies with larger sample sizes. Third, intersectionality originates in qualitatively and theoretically informed research. Thus, future qualitative research among women with multiple forms of disadvantages can explore the context specific stories.

## Conclusions

More than two-thirds of women had contact coverage of 4ANC visits and institutional delivery, and one in two women received PNC for mothers and newborns. The contact coverage of routine MNH visits was low among women with multiple forms of disadvantages. Intersectional analysis can be instrumental in identifying the women with intersectional (dis) advantages and could inform designing of contextual and targeted approaches to improve MNH. Thus, the actions towards UHC for MNH should start by addressing barriers of access among women with intersectional disadvantages, especially in resource-poor settings such as Nepal.

## Supplementary Information

**Additional file 1: Supplementary Figure S1.** Summary of sampling design used in the NDHS 2016**.** Table S1 Socioeconomic and geographic variables included examining the extent of inequities in MNH visits in Nepal, 2016.

## Data Availability

Data used in this study are publicly available secondary data obtained from the DHS **(**https://dhsprogram.com/data/available-datasets.cfm**)** program.

## Abbreviations

ANC: Antenatal Care
CoC: Continuum of Care
LMICs: Low and lower middle-income countries
MNH: Maternal and newborn health
NDHS: Nepal Demographic and Health Survey
PNC: Postnatal Care
SDIP: Safe Delivery Incentive Program
UHC: Universal health coverage
