# Supplementary material for: Intersectional (in) equities in contact coverage of maternal and newborn health services in Nepal: insights from a nationwide cross-sectional household survey
Source: BMC Public Health. 2021 Jun 9;21:1098. doi: 10.1186/s12889-021-11142-8 (PMC8190849; doi:10.1186/s12889-021-11142-8)
Supplement: Supplementary file 1 — Additional file 1: Supplementary Figure S1. Summary of sampling design used in the NDHS 2016. Table S1 Socioeconomic and geographic variables included examining the extent of inequities in MNH visits in Nepal, 2016. [file 12889_2021_11142_MOESM1_ESM.doc]

## Supplementary file

Figure 1: Summary of sampling design used in the NDHS 2016

Nepal: Seven provinces

Seven urban strata comprised of many clusters (wards) also called PSUs. Each ward comprised an average of 800 households.

Seven rural strata, each stratum comprised of clusters (wards) or primary sampling units (PSUs) also considered as Enumeration areas (EAs). Each ward (or EA) or PSU comprised an average of 104 households in rural areas.

If wards or PSUs had >200 households: segmented into sub-ward enumeration areas (EAs).

383 PSUs were randomly selected, one EA was selected from each PSU.

30 households per EAs

Included only 1,978 women who had a live birth two years preceding the survey.

There were 3,998 women who had a live birth five years preceding the survey.

From households selected 11,203 but 11,040 responded. From those households, of 13,089 women age 15-49 years identified for interview, 12,862 responded (response rate of 98%).

Table 1: Socioeconomic and geographic variables inlcudeded in the inequities of contact coverage of routine MNH visits in Nepal, 2016.

| **Variables** | **Categories** | **Descriptions** |
| --- | --- | --- |
| **Independent variables** |  |  |
| **Structural** |  |  |
| Wealth status[[1]](#footnote-2) | Poor (lowest two quintile) =0; Rich (upper three quintile) =1 | NDHS data had a variable wealth quintile, which was calculated based on scores generated from principal components analysis of households' assets (40 items). It was grouped into two categories: poor (poorest and poor; collectively called as lower two quintiles); and rich (middle, richer and richest collectively called as upper three quintiles) wealth status |
| Ethnicity | Disadvantaged=0; Advantaged=1 | Disadvantaged: Dalit, Muslims, and Terai caste, Janajatis disadvantaged) and advantaged: Brahmin/Chhetri, advantaged Janajatis) |
| Maternal education | Illiterate =0; illiterate=1 | Illiterate: Cannot read and write; literate who can read and write or attended school or higher-level education |
| **Intersectionality** |  |  |
| Marginalisation status of women | Poor and illiterate and disadvantaged ethnicity =0.  Poor and illiterate and advantaged ethnicity =1.  Poor and literate and disadvantaged ethnicity=2.  Rich and illiterate and disadvantaged ethnicity=3.  Poor and literate and advantaged ethnicity=4.  Rich and illiterate and advantaged ethnicity=5.  Rich and literate and disadvantaged ethnicity=6.  Rich and literate and advantaged ethnicity=7. | A composite categorisation of maternal education, ethnicity, and wealth status of women |
| **Intermediary** |  |  |
| Langauge | Nepali=0, Maithili=1, Bhojpuri=2, Others =3 (e.g., Newari, Tharu) | First language of respondents |
| Residence | Urban=0; Rural=1 | Municipalities are called urban, and remaining parts are called rural areas |
| Province | 1-7 provinces (province 1=0 as a reference province) | Now, provinces are numbered (to be named) |
| Region | Mountain=0, Hills=1, Terai=2 | Ecological region |
| **Outcome variables** |  |  |
| Three outcome variables across the CoC | Four ANC visits: 1= yes; 0=no.  Institutional delivery: 1= yes; 0=no.  Postnatal care of mothers and newborns within 48 hours of childbirth: 1= yes; 0=no. | Each outcome variable has binary categories: 1= yes; 0=no. |

1. Poor include lower 40% of wealth quintile while rich include upper 60% of wealth quintile. [↑](#footnote-ref-2)
